# Dietary habits in adolescent male and female handball players: the Swedish Handball Cohort

**DOI:** 10.1136/bmjsem-2023-001679

**Published:** 2023-12-21

**Authors:** Clara Onell, Eva Skillgate, Anna Melin, Henrik Källberg, Markus Waldén, Klara Edlund, Martin Hägglund, Pierre Côté, Martin Asker

**Affiliations:** 1 Department of Health Promotion Science, Sophiahemmet University, Stockholm, Sweden; 2 Unit for Intervention and Implementation Research in Worker Health, Institute of Environmental Medicine, Karolinska Institutet, Stockholm, Sweden; 3 Department of Sport Science, Linnaeus University, Växjö/Kalmar, Sweden; 4 Department of Public Health, Analysis and Data Management, The Public Health Agency of Sweden, Solna, Sweden; 5 Unit of Public Health, Department of Health, Medicine and Caring Sciences, Linköping University, Linköping, Sweden; 6 Capio Ortho Center Skåne, Malmö, Sweden; 7 Division of Psychology, Department of Clinical Neuroscience, Karolinska Institutet, Stockholm, Sweden; 8 Unit of Physiotherapy, Department of Health, Medicine and Caring Sciences, Linköping University, Linköping, Sweden; 9 Institute for Disability and Rehabilitation Research, Faculty of Health Sciences, University of Ontario Institute of Technology, Oshawa, Ontario, Canada; 10 Naprapathögskolan - Scandinavian College of Naprapathic Manual Medicine, Stockholm, Sweden

**Keywords:** Adolescent, Athlete, Sports and nutrition, Handball, Epidemiology

## Abstract

**Objectives:**

This cross-sectional study aimed to describe dietary habits in Swedish adolescent handball players and differences with respect to sex and school grade.

**Methods:**

Participants in the Swedish Handball Cohort answered a web-survey assessing adherence to sports nutrition recommendations for meal frequency and meal timing, and the Nordic Nutrition Recommendations (NNR) for fruits/vegetables and fish/seafood, food exclusions and use of dietary supplements. Differences with respect to sex and school grade were estimated with generalised linear models, generating prevalence ratios (PR) with 95% CIs.

**Results:**

A total of 1040 participants (16.6±0.9 years, 51% males) were included. Overall, 70% and 90%, respectively, met recommendations for meal frequency and meal timing, whereas adherence to recommended carbohydrate intake during training/game was met by 17%. Adherence to the NNR for fruits/vegetables and fish/seafood was met by 16% and 37%, respectively. Twenty-eight per cent reported using dietary supplements. Females reported lower frequency of meals, especially morning snacks (−0.6 days/week (95% CI −0.3 to −0.9)) and evening snacks (−0.8 days/week (95% CI −0.5 to –1.1)), higher prevalence of exclusions due to intolerances (PR 1.66 (95% CI 1.31 to 2.01)) and other reasons (PR 1.36 (95% CI 1.08 to 1.64)), higher adherence to the NNR for fruits/vegetables (PR 2.30 (95% CI 1.98 to 2.62)) and use of micronutrient supplements (PR 1.72 (95% CI 1.43 to 2.00)) compared with males. Only small differences were observed between school grades.

**Conclusions:**

Swedish adolescent handball players’ dietary habits are fairly in accordance with sports nutrition recommendations but not the NNR. Females appear to display more restrictive habits than males.

**WHAT IS ALREADY KNOWN ON THIS TOPIC**

- Challenges with meeting energy and nutrient requirements in athletes are well known and may result in serious implications for health and performance. Low adherence to dietary recommendations in adolescent and adult athletes has previously been reported, yet dietary habits in adolescent handball players have until recently been unknown.

**WHAT THIS STUDY ADDS**

- This study suggests that Swedish adolescent handball players’ dietary habits are in accordance with sports nutrition recommendations for meal frequency and meal timing but not carbohydrate intake during training/game, which may increase the risk of low carbohydrate availability during high-intensity activities. The Nordic Nutrition Recommendations for fruits/vegetables and fish/seafood, as an estimate of intake of micronutrients (eg, vitamins and minerals), are met to a low extent. Females report lower meal frequency, more exclusion of foods due to intolerances and other reasons, a higher intake of fruits/vegetables and use of micronutrient supplements compared with males, which might bring challenges with meeting energy requirements and, ultimately, impact health and performance.

**HOW THIS STUDY MIGHT AFFECT RESEARCH, PRACTICE OR POLICY**

- The knowledge gained from this study may be valuable for practitioners in youth team sports by identifying areas in need of nutritional education. Highlighting the importance of a proper energy and nutrient intake in adolescent sports settings brings sustainable health promotion for active adolescents.

## Introduction

Engaging in sports during youth brings fitness, social connections, better school achievements and improved mental health^1^ as well as healthy lifestyle habits later in life.^2 3^ A balanced dietary intake in young athletes is fundamental to support development in the transition to emerging adulthood as well as to sports performance and recovery.^4 5^ An insufficient energy and nutrient availability may compromise recovery and adaptation to training and increase the risk of injuries, delayed puberty and poor bone health.^5^

Dietary requirements get increasingly important with increased training volume and the substantial energy requirement might be practically difficult to fulfil.^6^ Intense training may have an appetite-suppressing effect, athletes may eat hours before training to prevent gastrointestinal distress and training schedules, school obligations and travelling may limit food availability and time to eat.^6^ Also, athletes are susceptible to peer pressure and appearance ideals, which might lead to disordered eating and mental health problems.^7^

Carbohydrate intake is a prerequisite for high-intensity exercise to ensure glycogen stores and substrate.^8^ Although nutrient-dense foods such as whole grains and vegetables are recommended as the main source of carbohydrates for health promotion in the general population,^9^ energy-dense and low-fibre alternatives^8^ including pasta, cereals and dried fruit, as well as refined sugars, are recommended for athletes to ensure adequate energy and carbohydrate availability.^10^ This is important prior to, during and after high-intensity and long training sessions, and in developing athletes.^11^ To meet these requirements, athletes are recommended to have a balanced diet including a variety of foods, a frequent meal pattern with 4-6 meals/day, including carbohydrate and protein rich foods in concert with training.^6^

Handball is a popular team-sport with an estimated five million youth players. The sport’s intermittent nature with alternation between offensive and defensive elements^12^ impose demands on anaerobic and aerobic energy systems, in which glycogen is the main energy substrate.^13^ Adult handball players have been reported to not meet nutrition recommendations.^14–17^ Studies on adolescent handball players’ dietary habits are scarce and no previous studies have, to the best of our knowledge, examined differences with respect to sex and age. This study aimed to describe dietary habits in Swedish adolescent handball players and differences with respect to sex and school grade.

## Methods

### The Swedish Handball Cohort

This cross-sectional study is based on the Swedish Handball Cohort (SHC) aiming to investigate implementation of injury prevention exercise programmes and identify risk factors for injuries in youth handball. The SHC builds on the Karolinska Handball Study with similar methodology.^18^ The inclusion criterium was enrolment to a handball-profiled secondary school and the exclusion criterium was inability to understand Swedish. Participants answered a web-survey in the fall season 2020 or 2021 including questions related to playing handball (eg, playing position, playing level) and dietary habits. The survey was administered automatically through the platform Easy Teams (lynes Technologies Sweden AB). Adolescent handball players were invited with a link to the baseline survey through their school handball coaches, with a reminder if they had not answered. Participants, and legal guardians for participants below the age of 15 years, gave their written informed consent prior to filling out the survey. This study was conducted in accordance with STrengthening the Reporting of OBservational Studies in Epidemiology statements. The protocol complied with local legal requirements as well as the Declaration of Helsinki for research involving human subjects.

### Dietary habits

#### Meal frequency

Meal frequency was assessed as intake of breakfast, morning snack, lunch, afternoon snack, dinner, evening snack and other snacks (eg, recovery snack) in 0-7 days/week for each meal. This question has previously been used to assess meal frequency in young athletes.^19^

#### Meal timing

Meal timing was assessed with questions about having a meal within 3 hours before and within 1 hour after training, respectively. The questions’ readability was evaluated in a group of frequently physically active students generating some rephrasing. Response options included eating a main meal, a snack or no meal before/after training. For participants answering no meal, a question about the reason for this was asked with response options limit of time, lack of appetite, practical issues or other (free text). Intake and frequency of fluid and/or food other than water (such as sport drinks, gainers, gels, bars) during training/game was assessed as a measure of carbohydrate intake, with 14 response options ranging from never or <1 time/month to 4 times/day or more.

#### Adherence to the Nordic Nutrition Recommendations

Intake of micronutrients was evaluated in accordance with the Nordic Nutrition Recommendations (NNR) with a food frequency questionnaire.^20^ Habitual intake of vegetables/legumes, fruits/berries, potatoes and fish/seafood during the past 12 months was assessed, with 14 response options ranging from never or <1 time/month to 4 times/day or more. Participants were asked to only report portions (eg, not if only one slice of cucumber). Adhering to the NNR was defined as eating vegetables/legumes, fruits/berries and/or potatoes (ie, fruits/vegetables) ≥4 times/day (~500 g/day) and fish/seafood ≥2 times/week.

#### Food exclusions

Adherence to a diet was evaluated with a question developed for this study, with response options omnivore, vegetarian, vegan or other. For those answering other diet, a question regarding what type of diet was asked (free text). Exclusion of foods due to intolerances (yes/no) or other reasons (yes/no) was assessed as well as what type of foods excluded (free text).

#### Use of dietary supplements

Use of dietary supplements was assessed with a question about whether the participants used any supplements (yes/no) and, if so, which type of dietary supplement (free text) and how often with 14 response options ranging from never or <1 time/month to 4 times/day or more.

### Statistical analyses

Meal frequency was presented as average days/week for each meal and mean differences with 95% CIs to assess potential differences with respect to sex. Meal timing, food exclusions, adherence to the NNR and use of dietary supplements were presented as proportions and prevalence ratios (PR) with 95% CI for differences with respect to sex and school grade, estimated using generalised linear models with a negative binomial link function. Sex and school grade were included in each model when comparing dietary habits between sex and school grade, respectively (eg, when analysing differences with respect to sex, school grade was adjusted for and vice versa). Statistical analyses were performed using RStudio V.1.3.1093.

### Patient and public involvement

This is an observational study without interventions and patients. The SHC was designed due to a high injury incidence in youth handball. Since nutrition plays a major role for health, performance and recovery, assessment of dietary habits was performed in this population. The areas of dietary habits to measure were decided based on clinical experience, involving practitioners as well as active youths to ensure relevant questions about dietary habits targeting the intended population. The knowledge gained from this study will be disseminated through written reports to handball coaches in each included secondary school with recommendations how dietary habits could be addressed.

## Results

Thirty-six handball-profiled secondary schools with 1578 potential participants were invited to answer the baseline survey. Nineteen schools with a total of 1040 adolescent handball players (66%) agreed to participate (mean age 16.6±0.9 years, 51% males). Most participants were enrolled during first grade and backcourt players on a regional level. Mean weekly time playing handball was 12 hours. Participant characteristics are presented in table 1.

**Table 1:** Participant characteristics

Variables	All (n=1040)	Male (n=532)	Female (n=508)
Age, mean years±SD	16.6±0.9	16.6±0.9	16.6±0.9
Years of playing handball, mean±SD	9.4±2.1	9.3±2.3	9.5±2.0
School grade, n (%)			
1st year	498 (48)	266 (50)	232 (46)
2nd year	295 (28)	149 (28)	146 (29)
3rd year	247 (24)	117 (22)	130 (26)
Playing level past season, n (%)			
Regional level*	805 (77)	403 (76)	402 (79)
National level†	235 (23)	129 (24)	106 (21)
Playing position past season, n (%)			
Goalkeeper	144 (14)	70 (13)	74 (15)
Backcourt player	541 (52)	264 (50)	277 (55)
Line player	141 (14)	76 (14)	65 (13)
Wing player	214 (21)	122 (23)	92 (18)
Games past season, mean n/week±SD	1.1±0.8	1.1±0.8	1.1±0.8
On-court training past season, mean h/week±SD	6.2±2.4	6.2±2.4	6.2±2.4
Off-court training past season, mean h/week±SD	4.2±2.2	4.2±2.2	4.2±2.2
Participation in other forms of physical activities (eg, other sports) past season, n (%)	251 (24)	166 (31)	85 (17)

*Played for a local club or district team the past season.

†Played for a national youth team or summoned to a national camp the past season.

### Meal frequency

Meal frequency and mean differences between sex are presented in table 2. Seventy per cent reported eating 4-6 meals/day. Mean days/week of eating main meals (breakfast, lunch and/or dinner) were 6–7 days/week (range 0–7) and in-between snacks 3.5–5.0 (range 0–7) days/week. Meal frequency was lower for females than males for all meals, with the biggest difference in morning snack, evening snack and other snacks.

**Table 2:** Meal frequency in mean days/week±SD for all participants and stratified by sex

Meal intake	All (n=1040)	Male (n=532)	Female (n=508)	Mean difference (95% CI)
Breakfast	6.3±1.4	6.4±1.3	6.3±1.5	−0.1 (−0.1 to −0.3)
Morning snack	3.5±2.3	3.8±2.3	3.2±2.2	−0.6 (−0.3 to −0.9)
Lunch	6.7±0.7	6.9±0.5	6.6±0.9	−0.3 (−0.2 to −0.4)
Afternoon snack	5.0±1.9	5.1±2.0	4.9±1.9	−0.2 (−0.0 to −0.4)
Dinner	6.9±0.7	6.9±0.7	6.8±0.8	−0.1 (−0.0 to −0.2)
Evening snack	4.6±2.2	5.0±2.1	4.2±2.2	−0.8 (−0.5 to −1.1)
Other snack 1	1.9±2.2	2.2±2.4	1.5±1.9	−0.7 (−0.4 to −1.0)
Other snack 2	1.2±1.9	1.4±2.1	1.0±1.6	−0.4 (−0.2 to −0.6)

### Meal timing

Meal timing and comparison with respect to sex and school grade is presented in online supplemental appendix tables 1 and 2. Eighty-eight per cent reported having a meal within 3 hours before training and 90% within 1 hour after. Of these, 54% and 76% reported having a main meal before morning and evening training, respectively (not in table). The most common reasons for not eating before/after training were lack of appetite and limit of time. Intake of carbohydrates during training/game was reported by 17%. Among these, 6% reported daily intake, 64% weekly intake and 30% monthly intake. There were no differences with respect to sex or school grade.

10.1136/bmjsem-2023-001679.supp1 Supplementary data

### Adherence to the Nordic Nutrition Recommendations

Adherence to the NNR for fruits/vegetables and fish/seafood intake was 16% and 37%, respectively. Comparison with respect to sex and school grade is presented in table 3, showing a higher adherence to fruits/vegetables recommendations in females compared with males (PR 2.30 (95% CI 1.98 to 2.62)). No difference was observed with respect to school grade. Likewise, there was no difference with respect to sex or school grade for fish/seafood recommendations.

**Table 3:** Adherence to the Nordic Nutrition Recommendations n (%), and comparison between sex and school grade

	Eating fruits/vegetables ≥4 times/day, n (%)	Prevalence ratio of eating fruits/vegetables ≥4 times/day (95% CI)
All (n=1040)	169 (16)	
Sex		
Male (n=532)	53 (10)	1 (Ref)*
Female (n=508)	116 (23)	2.30 (1.98 to 2.62)
School grade		
1st (n=498)	83 (17)	1 (Ref)†
2nd (n=295)	48 (16)	0.95 (0.60 to 1.30)
3rd (n=247)	38 (15)	0.88 (0.51 to 1.25)

	Eating fish/seafood ≥2 times/week, n (%)	Prevalence ratio of eating fish/seafood ≥2 times/week (95% CI)
All (n=1040)	386 (37)	
Sex		
Male (n=532)	195 (37)	1 (Ref)*
Female (n=508)	191 (38)	1.03 (0.83 to 1.23)
School grade		
1st (n=498)	188 (38)	1 (Ref)†
2nd (n=295)	110 (37)	0.99 (0.77 to 1.21)
3rd (n=247)	88 (36)	0.94 (0.70 to 1.18)

*Adjusted for school grade.

†Adjusted for sex.

### Food exclusions

Food exclusions are presented in table 4. Ninety-seven per cent reported following an omnivore diet and 2% reported following a vegetarian diet. Thirteen per cent reported having food intolerances where lactose, dairy, gluten, nuts and drupes were most common. Females reported having food intolerances to a larger extent than males (PR 1.66 (95% CI 1.31 to 2.01)) and participants in third school grade reported having food intolerances to a larger extent compared with first school grade (PR 1.67 (95% CI 1.29 to 2.05)).

**Table 4:** Food exclusions n (%), and comparison between sex and school grade

	Food exclusion due to intolerance, n (%)	Prevalence ratio of food intolerance (95% CI)
All (n=1040)	136 (13)	
Sex		
Male (n=532)	52 (10)	1 (Ref)*
Female (n=508)	84 (17)	1.66 (1.31 to 2.01)
School grade		
1st (n=498)	55 (11)	1 (Ref)†
2nd (n=295)	34 (12)	1.03 (0.61 to 1.45)
3rd (n=247)	47 (19)	1.67 (1.29 to 2.05)

	Food exclusion due to other reasons than intolerance, n (%)	Prevalence ratio of food exclusion (95% CI)
All (n=1040)	205 (20)	
Sex		
Male (n=532)	90 (17)	1 (Ref)*
Female (n=508)	115 (23)	1.36 (1.08 to 1.64)
School grade		
1st (n=498)	109 (22)	1 (Ref)†
2nd (n=295)	62 (21)	0.95 (0.65 to 1.25)
3rd (n=247)	34 (14)	0.62 (0.25 to 0.99)

*Adjusted for school grade.

†Adjusted for sex.

Excluding food due to other reasons was reported by 20% of the participants. Most common food groups to exclude were seafood, fruits/vegetables, red meat and energy-dense foods (ie, candy, fast food, sugar-sweetened beverages). Females reported excluding foods due to other reasons than intolerances to a larger extent than males (PR 1.36 (95% CI 1.08 to 1.64)). Participants in third school grade reported excluding food items to a less extent compared with those in first school grade (PR 0.62 (95% CI 0.25 to 0.99)).

### Use of dietary supplements

Use of dietary supplements was reported by 28% as presented in online supplemental appendix table 3. Of these, 44% reported using supplements daily, 47% weekly and 9% monthly. The most common supplements were protein/creatine, omega-3 fatty acids, vitamin C, vitamin D, iron and magnesium. Using micronutrient supplements was more common in females than males (PR 1.72 (95% CI 1.43 to 2.00) whereas using protein/creatine supplements was less common in females (PR 0.06 (95% CI 0.00 to 1.06)) compared with males. Using protein/creatine supplements was more common in third school grade compared with first school grade (PR 1.42 (95% CI 0.95 to 1.89)).

## Discussion

This study found that dietary habits in Swedish adolescent handball players are somewhat in accordance with nutrition recommendations. Most participants reported eating 4-6 meals/day and time meals in concert with training. In contrast, adherence to recommendation of carbohydrate intake during training/game was poor, which is in accordance with previous studies showing an insufficent carbohydrate intake in team-sport athletes.^15^ Also, the NNR for fruits/vegetables and fish/seafood were adhered to only to a low extent, which is in line with studies on non-athlete^21^ and athlete^17^ adolescent populations, including handball players. Adolescents are recommended to meet minimal recommended intakes of food groups such as fruits, vegetables and fish to ensure a sufficient intake of micronutrients, omega-3 fatty acids and fibre, among others, and gain their health-promoting benefits.^9^ Lack of appetite and limit of time were the most common reasons for not eating in concert with training, which are known concerns for meeting energy requirements in athletes.^6 11^ Having an insufficient energy intake might be intentional (eg, dieting) or unintentional (eg, suppressed post-training appetite), yet result in problematic low energy availability^22^ which may manifest as relative energy deficiency in sports (REDs). REDs is a serious syndrome impacting for example, reproductive, metabolic, cardiovascular and mental health.^23^ Low energy availability and REDs have historically been mostly discussed in relation to weight-sensitive sports, however, a recent study shows that these conditions are prevalent also in female handball players.^24^ Although energy and nutrient requirements can be met through well-balanced meals,^9^ timing of carbohydrate and protein intake is key for enhancing adaptive responses to exercise.^6^ This is particularly important in athletes with a high training load as in this study, with a weekly training volume of on average 12 hours. A high meal frequency should be encouraged and timed with training as it facilitates the possibility of meeting energy and nutrient requirements,^6^ improves within-day energy balance^25^ and ensures recovery and restoration of glycogen.^6^

More than one-fourth reported using dietary supplements, which is higher than previously reported in adult handball players.^26 27^ Athletes and non-athletes living in high-latitude countries may be at risk of vitamin D deficiency, and hence benefit from supplementation.^9^ Vitamin C supplementation, on the other hand, was frequently reported although deficiency is very rare, and requirements are easily fulfilled from the diet.^28^ Moreover, iron was one of the most used supplements, especially in females. The risk of iron deficiency is high in female athletes through loss via menstrual bleeding, sweating and erythrocyte destruction.^29^ Iron intake below the recommended dietary allowance and low ferritin levels have previously been reported in female handball players.^30^ Unfortunately, it is not known whether the athletes in this study used iron supplementation ordinated by a physician, but it is important to emphasise that supplementation without deficiency should be avoided as it might result in toxicity.^9^ It is important to mention that dietary supplements cannot overcome the deficits of poor nutrition,^28^ and an unduly focus on supplements might lead to less focus on having healthy dietary habits, as well as increase the risk of unintentional doping.^31^ The priority for health, growth and performance in adolescent athletes is to ensure energy intake matching the level of physical activity, to facilitate nutrient intakes. Focus should, therefore, be on adequate dietary habits.

In this study, almost all participants reported adhering to an omnivore diet, which is in line with reports of non-athlete Swedish adolescents.^21^ The results differ from previous studies reporting a higher prevalence of adhering to a vegetarian diet in adolescent athletes in several countries,^32^ which could be explained by cultural differences in non-Western populations. The most reported food intolerances were lactose, dairy, gluten, nuts and drupes, similar to intolerances in female endurance athletes.^33^ The prevalence of food intolerances was higher in this study, and it is not clear to what extent these were medically rationalised or adopted based on a perception of health benefits. Adherence to special diets to prevent gastrointestinal distress by excluding groups of carbohydrates has previously been reported in athletes^34 35^ but was not observed in this study. Excluding foods due to other reasons than an intolerance might make it even more difficult to meet the substantial requirements in an already vulnerable group at risk of inadequate intakes.^14–16^

Females reported lower frequency of all meals, higher prevalence of food exclusions, higher intake of fruits/vegetables and use of micronutrient supplements compared with males. The lower intake of fruits and vegetables in males has earlier been reported,^17 21^ although both males and females overall failed to meet the NNR in this study. Furthermore, type of dietary supplements differed between females and males in this study. The more frequent use of protein/creatine supplementation in males could be related to different views on physical attributes expected in male and female athletes, where concerns about weight and body shape are more common in females and concerns about insufficient muscularity is more common in males.^36^ The results of this study indicate that females may display more restrictive dietary habits compared with males, with a potential lower energy intake and increased risk of REDs.

The prevalence of food intolerances was higher in higher school grades, which is surprising given that food intolerances often are less common with increased age. At the same time, food exclusions due to other reasons than intolerances decreased with higher school grades, while the use of protein/creatine supplements increased with higher school grades. These findings could, hypothetically, be explained by a higher interest in nutrition with increased age resulting in reporting exclusion of certain food groups due to a perceived health effect as intolerances.^35^

### Methodological discussion

This is the largest known study with a representative sample of adolescent handball players with a high inclusion rate of participants from the majority of the Swedish handball-profiled secondary schools. The results are likely generalisable to other team-sport athletes in similar cultural contexts and training schedules. Besides including a validated food frequency questionnaire,^20^ questions to deepen the knowledge about dietary habits in a bigger context were developed, including measures that describe the possibility to meet energy and nutrient requirements without focusing on assessing actual nutrient and energy intakes through objective measures. This is considered a strength since self-registered data on energy and nutrient intakes is demanding and generally reflects a limited number of days, not capturing the habitual intake, and one of the reasons why nutritional epidemiology has shifted focus from assessing single nutrients to instead evaluating overall dietary habits.^37^

### Limitations

This study is not without limitations. The risk of misclassification would likely be lower using validated measures of dietary habits specifically targeting populations of young team-sport athletes. It is also possible that the differences in dietary habits with respect to sex are explained by other systematic differences in these groups (eg, socioeconomic status). Furthermore, the dietary habits assessed in this study are a proxy for nutrient and energy intakes, but do not reveal actual intakes, which fulfilment is the primary consideration as an athlete. Although assessing nutritional intakes in observational studies often are challenging (eg, due to underreporting), the knowledge about dietary habits in adolescent handball players would be further deepened if this study also included accurate measures on energy and nutrient intakes, as well as anthropometric data (eg, body composition) and energy expenditure to quantify energy requirements. Another limitation is the absence of measurement of eating attitudes including disordered eating and eating disorders as well as symptoms of REDs. Given that these problems are common in young athletes, and not yet studied in adolescent handball players, this knowledge would further deepen the understanding about the most relevant questions related to dietary habits to address in this population, especially with regard to the sex differences observed where females display potentially more restrictive dietary habits. In future studies, health aspects such as injury prevalence, menstrual dysfunction and sleep impairments may act as proxies for energy availability and REDs in adolescent athletes and can be used to further deepen the knowledge about dietary habits and athletes’ health. Finally, dietary habits were self-reported which is a limitation given that health behaviours tend to be reported in accordance with social desirability.

### Clinical implications

The knowledge gained from this study may be valuable for identifying areas in need of nutritional education/interventions in handball-profiled secondary schools, as well as similar contexts with adolescent team-sport athletes, to improve health in active adolescents. This is also in line with the United Nations’ Agenda 2030, aiming to ensure healthy lives and promote well-being for all at all ages. To further inform practitioners about the importance of dietary habits in youth handball, prospective studies investigating how the findings of this study are related to adolescent handball players’ health and performance with regards to injury risk are warranted.

## Conclusions

Swedish adolescent handball players’ dietary habits regarding meal frequency and timing seem to be in accordance with sports nutrition recommendations, while adherence to recommendations for intake during training/game and the NNR for fish/seafood and fruits/vegetable intake is low. Females seem to display more restrictive dietary habits than males.

## Data Availability

Data are available on reasonable request.
