# Supplementary material for: Dietary habits in adolescent male and female handball players: the Swedish Handball Cohort
Source: BMJ Open Sport Exerc Med. 2023 Dec 21;9(4):e001679. doi: 10.1136/bmjsem-2023-001679 (PMC10749036; doi:10.1136/bmjsem-2023-001679)
Supplement: Supplementary data [file bmjsem-2023-001679supp001.pdf]

Appendix table 1. Meal timing n (%), and comparison between sex and school grade.

	Eating within 3 hours before handball training, n (%)	Prevalence ratio for eating within 3 hours before handball training (95% CI)
All (n=1040)	916 (88)	
Sex		
Male (n=532)	487 (92)	1 (Ref)*
Female (n=508)	429 (84)	0.92 (0.79, 1.05)
School grade		
1st (n=498)	444 (89)	1 (Ref)ǂ
2nd (n=295)	262 (89)	1.00 (0.86, 1.13)
3rd (n=247)	210 (85)	0.96 (0.82, 1.10)
	**Eating within 1 hour after handball training, n (%)**	**Prevalence ratio for eating within 1 hour after handball training (95% CI)**
All (n=1040)	939 (90)	
Sex		
Male (n=532)	492 (92)	1 (Ref)*
Female (n=508)	447 (88)	0.96 (0.83, 1.09)
School grade		
1st (n=498)	469 (94)	1 (Ref)ǂ
2nd (n=295)	260 (88)	0.94 (0.81, 1.07)
3rd (n=247)	210 (85)	0.91 (0.77, 1.05)

*Adjusted for school grade.

ǂAdjusted for sex.

Onell C, *et al. BMJ Open Sp Ex Med* 2024; 9:e001679. doi: 10.1136/bmjsem-2023-001679

Appendix table 2. Intake during training/game n (%), and comparison between sex and school grade.

	Intake during training/game, n (%)	Prevalence ratio for intake during training/game (95% CI)
All (n=1040)	177 (17)	
Sex		
Male (n=532)	101 (19)	1 (Ref)*
Female (n=508)	76 (15)	0.89 (0.59, 1.19)
School grade		
1st (n=498)	95 (19)	1 (Ref)ǂ
2nd (n=295)	41 (14)	0.99 (0.63, 1.35)
3rd (n=247)	41 (17)	0.99 (0.63, 1.35)

*Adjusted for school grade.

ǂAdjusted for sex.

Appendix table 3. Use of dietary supplements n (%), and comparison between sex and school grade.

	Use of dietary supplements, n (%)	Prevalence ratio for use of dietary supplements (95% CI)
All (n=1040)	290 (28)	
Sex		
Male (n=532)	157 (30)	1 (Ref)*
Female (n=508)	133 (26)	0.89 (0.66, 1.12)
School grade		
1st (n=498)	140 (28)	1 (Ref)ǂ
2nd (n=295)	82 (28)	0.99 (0.73, 1.25)
3rd (n=247)	68 (28)	0.99 (0.71, 1.27)
	**Use of micronutrient supplements, n (%)**	**Prevalence ratio for use of micronutrient supplements (95% CI)**
All (n=290)	199 (69)	
Sex		
Male (n=157)	76 (48)	1 (Ref)*
Female (n=133)	118 (89)	1.72 (1.43, 2.00)
School grade		
1st (n=140)	95 (68)	1 (Ref)ǂ
2nd (n=82)	58 (71)	1.06 (0.76, 1.36)
3rd (n=68)	46 (68)	1.03 (0.72, 1.34)
	**Use of protein/creatine supplements, n (%)**	**Prevalence ratio for use of protein/creatine supplements (95% CI)**
All (n=290)	81 (28)	
Sex		
Male (n=157)	77 (49)	1 (Ref)*
Female (n=133)	4 (3)	0.06 (0.00, 1.06)
School grade		
1st (n=140)	36 (26)	1 (Ref)ǂ
2nd (n=82)	18 (22)	0.82 (0.27, 1.37)
3rd (n=68)	27 (40)	1.42 (0.95, 1.89)

*Adjusted for school grade.

ǂAdjusted for sex.
